# GMP-compliant production of [^68^Ga]Ga-NeoB for positron emission tomography imaging of patients with gastrointestinal stromal tumor

**DOI:** 10.1186/s41181-021-00137-w

**Published:** 2021-07-06

**Authors:** Marc Pretze, Laura Reffert, Steffen Diehl, Stefan O. Schönberg, Carmen Wängler, Peter Hohenberger, Björn Wängler

**Affiliations:** 1 Department of Nuclear Medicine, University Hospital Carl Gustav Carus, TU Dresden, Fetscherstr. 74, 01307 Dresden, Germany; 2 Molecular Imaging and Radiochemistry, Department of Clinical Radiology and Nuclear Medicine, Medical Faculty Mannheim of Heidelberg University, Theodor-Kutzer-Ufer 1-3, 68167 Mannheim, Germany; 3 Department of Clinical Radiology and Nuclear Medicine, University Medical Center Mannheim, Mannheim, Germany; 4 Biomedical Chemistry, Department of Clinical Radiology and Nuclear Medicine, Medical Faculty Mannheim of Heidelberg University, Mannheim, Germany; 5 Division of Surgical Oncology and Thoracic Surgery, University Medical Center Mannheim, Mannheim, Germany

**Keywords:** [^68^Ga]Ga-NeoB, GRPR, GMP, PET/CT, Metastatic GIST

## Abstract

**Background:**

[^68^Ga]Ga-NeoB is a novel DOTA-coupled Gastrin Releasing Peptide Receptor (GRPR) antagonist with high affinity for GRPR and good in vivo stability. This study aimed at (1) the translation of preclinical results to the clinics and establish the preparation of [^68^Ga]Ga-NeoB using a GMP conform kit approach and a licensed ^68^Ge/^68^Ga generator and (2) to explore the application of [^68^Ga]Ga-NeoB in patients with gastrointestinal stromal tumors (GIST) before and/or after interventional treatment (selective internal radiotherapy, irreversible electroporation, microwave ablation).

**Results:**

Validation of the production and quality control of [^68^Ga]Ga-NeoB for patient use had to be performed before starting the GMP production. Six independent batches of [^68^Ga]Ga-NeoB were produced, all met the quality and sterility criteria and yielded 712 ± 73 MBq of the radiotracer in a radiochemical purity of > 95% and a molar activity of 14.2 ± 1.5 GBq/μmol within 20 min synthesis time and additional 20 min quality control. Three patients (2 females, 1 male, 51–77 yrs. of age) with progressive gastrointestinal stromal tumor metastases in the liver or peritoneum not responsive to standard tyrosine kinase inhibitor therapy underwent both [^68^Ga]Ga-NeoB scans prior and after interventional therapy. Radiosynthesis of ^68^Ga-NeoB was performed using a kit approach under GMP conditions. No specific patient preparation such as fasting or hydration was required for [^68^Ga]Ga-NeoB PET/CT imaging. Contrast-enhanced PET/CT studies were performed. A delayed, second abdominal image after the administration of the of [^68^Ga]Ga-NeoB was acquired at 120 min post injection.

**Conclusions:**

A fully GMP compliant kit preparation of [^68^Ga]Ga-NeoB enabling the routine production of the tracer under GMP conditions was established for clinical routine PET/CT imaging of patients with metastatic GIST and proved to adequately visualize tumor deposits in the abdomen expressing GRPR. Patients could benefit from additional information derived from [^68^Ga]Ga-NeoB diagnosis to assess the presence of GRPR in the tumor tissue and monitor antitumor treatment.

**Supplementary Information:**

The online version contains supplementary material available at 10.1186/s41181-021-00137-w.

## Introduction

Gastrointestinal stromal tumors (GIST) are rare soft tissue mesenchymal tumors that occur in the gastrointestinal tract and are thought to be derived from the cells of Cajal, which conduct intestinal peristalsis (El-Menyar et al., 2017; Schaefer et al., 2017). For GISTs with a high risk of developing metastases, early detection with high sensitivity and by non-invasive methods would be an important improvement to allow for immediate treatment and to monitor or predict of the efficacy of therapy, which is currently mainly influenced by assessing the type of mutations in *KIT* or *PDGFRA* gene (Joensuu et al., 2017).

In this regard, morphological and functional imaging methods may be important for detection, staging and follow-ups of GIST-patients undergoing therapy. Computed tomography (CT) is the most frequently used morphological imaging procedure, although it lacks sensitivity and/or specificity. Functional imaging with positron emission tomography (PET) using 2-deoxy-2-[^18^F]fluoro-d-glucose combined with computed tomography ([^18^F]FDG PET/CT) is the most commonly used nuclear medicine functional imaging modality in clinic routine. It has shown to be advantageous over morphological imaging procedures alone when assessing therapy response. However, earlier studies using [^18^F]FDG PET/CT for GIST detection reported only the low/moderate sensitivity (Antoch et al., 2009). Hence, more accurate, specific and sensitive non-invasive diagnostic tools visualizing GIST are needed.

The gastrin-releasing peptide receptor (GRPR), also called bombesin receptor 2 (BB2) (Pooja et al., 2019), is a target for noninvasive PET imaging of various types of cancer (Liolios et al., 2018; Baratto et al., 2020). Since overexpression of the GRPR has been reported in various cancer types, e.g. prostate cancer (Markwalder & Reubi, 1999), breast cancer (Halmos et al., 1995), gastrointestinal stromal tumors (GISTs) (Reubi et al., 2004) and other tumors (Reubi et al., 2002). Targeting the GRP receptor with radioligands has a significant impact on the specific and sensitive detection and treatment of GRPR-expressing tumors (Cornelio et al., 2007). Bombesin (BBN) is a peptide with high affinity to the GRPR (Reubi, 2003; Smith et al., 2003). Therefore, various radiolabeled bombesin-based peptide ligands have been extensively used to target GRPR-expressing tumors such as GISTs (Reubi et al., 2004; Gonzalez et al., 2008; Dimitrakopoulou-Strauss et al., 2007). Derived from BBN, the truncated BBN_7–14_ sequence was developed, showing nearly the same affinity to GRPRs but with higher stability than bombesin. Hence, BBN_7–14_ has been used for the development of various radiopharmaceuticals with positron emitters such as ^18^F (Richter et al., 2013), ^64^Cu (Rogers et al., 2003) and ^68^Ga (Schuhmacher et al., 2005) for PET, with gamma emitters such as ^99m^Tc (Baidoo et al., 1998) for single photon emission computed tomography (SPECT), and with radionuclides such as ^90^Y (Zhang et al., 2004), ^111^In (Breeman et al., 1999) and ^177^Lu (Chatalic et al., 2016a) for endoradiotherapy. Additionally, optimization of the stability and affinity of the bombesin analogs has been explored by changing l- to d-amino acids (Chatalic et al., 2016b), utilizing triazole backbones in the peptide (Valverde et al., 2014) and applying multimerization (Lindner et al., 2014; Pretze et al., 2018).

Recently, [^68^Ga]Ga-NeoB (formerly known as NeoBOMB1) was developed, a novel 1,4,7,10-tetraazacyclododecane-1,4,7,10-tetra acetate (DOTA)-coupled bombesin-based GRPR antagonist, which showed a high affinity for GRPR (IC_50_ = 1.17 ± 0.06 nM (Nock et al., 2017)) and high tumor uptake in preclinical studies in a xenograft mouse model (30.7 ± 3.9%ID/g 4 h post injection (p.i.) in PC3 tumor-bearing mice) accompanied by an good in vivo stability (5 min p.i. > 95% intact, 30 min p.i. > 90% intact) (Nock et al., 2017). In preclinical studies the labeling method for [^68^Ga]Ga-NeoB proved to be high yielding and stable (Pretze et al., 2021; Lau et al., 2019). As known from the first clinical study, [^68^Ga]Ga-NeoB might have significant impact on the detection and treatment of GRPR expressing tumors such as like GIST (Gruber et al., 2020). Labeled with therapeutic radionuclides the peptide NeoB could also be useful for the treatment of imatinib-resistant GIST (Baratto et al., 2020).

The purpose of our study was to explore the applicability of [^68^Ga]Ga-NeoB for the determination of the status of GIST in patients with different GRPR expression levels confirmed by previous biopsies of those lesions. Furthermore, the GMP compliant production of [^68^Ga]Ga-NeoB was established in our good manufacturing practice (GMP) environment including risk management, installation qualification (IQ), operation qualification (OQ) and validation of the process in six independent productions of [^68^Ga]Ga-NeoB following most recent guidelines (Todde et al., 2017; Gillings et al., 2020).

In addition, the applicability of [^68^Ga]Ga-NeoB for visualization of GIST metastases before and/or after selective and patient-oriented specific interventional therapy (selective internal radiotherapy (SIRT), irreversible electroporation (IRE), microwave ablation (MWA)) was assessed.

## Methods

### Radiochemistry

The ^68^Ge/^68^Ga-generator holds a marketing authorization and was purchased from Eckert&Ziegler (1.85 GBq, GalliaPharm, Eckert & Ziegler, Berlin, Germany). The NeoB radiolabeling kit was received from Advanced Accelerator Applications S.A. (AAA). The experimental kit consisted of a vial containing the lyophilized NeoB precursor and additives and a second vial containing the reaction buffer. For automated generator elution, we used an automated synthesis module (Scintomics GRP, Fürstenfeldbruck, Germany) together with a cartridge kit for ^68^Ga-radiolabeling using this module (SC-103, ABX, Radeberg, Germany). Analytical (radio-)HPLC was performed on a Shimadzu HPLC system (Nakagyo-ku, Kyōto, Japan), equipped with a reverse phase column (Merck LiChrospher 100 RP-18; 125 × 3 mm), a UV-diode array detector (254 nm) and a scintillation radiodetector (Pomo, Elysia-Raytest, Straubenhardt, Germany). The solvent system used was a gradient of acetonitrile:water (containing 0.1% TFA) (0–20 min: 10–90% acetonitrile). [^18^F]FDG was commercially obtained (Life Radiopharma f-con GmbH, Germany). Thin-layer chromatography was performed with ITLC-SG strips (Agilent) in 1 M NH_4_Ac:MeOH 1:1 and a TLC scanner (Raytest). The pH was acquired by a QuantoFix Relax reflection photometer with the corresponding pH test strips 5.5 × 85 mm pH-Fix 2.0–9.0 (Macherey Nagel, Feucht, Germany). The endotoxinlevel was determined by using an Endosafe unit (Charles River, Wilmington, MA, USA). All the equipment received an IQOQ by external companies for GMP-compliant application.

### ^68^Ga-radiolabeling

The whole radiotracer production was performed inside a hot cell isolator (cleanroom class A, ITD, Dresden-Rossendorf, Germany) under GMP conditions 5 mL 0.1 M suprapur HCl were automatically eluted through the ^68^Ge/^68^Ga-generator and through a sterile filter (Millex-GS 0.22 μm, SLGSV255F, Millipore) directly into the reaction vial containing the NeoB precursor (50 ± 5 μg, 31.7 ± 0.6 nmol), resulting in a total reaction mixture volume of 3 mL containing 1100 ± 100 MBq ^68^Ga. Subsequently, the kit labeling buffer (0.50–0.55 mL 1 M formic acid with gentisic acid, pH 5) was added. Silicon-coated cannulas (0.6 × 60 mm, Sterican, B. Braun) were used throughout the whole synthesis process. Radiolabeling was performed at pH 3.6–4.0 for 7–10 min at 89 °C inside the reaction vial in a heating block (95 °C). The resulting solution contained 712 ± 73 MBq [^68^Ga]Ga-NeoB in a radiochemical purity (RCP) of 96–99%, as confirmed by radio-thin-layer chromatography (radio-TLC) and radio-HPLC (*t*_R_ = 9.7 min).

### Quality control under GMP conditions for the patient use

The quality control (QC) of the injectable radiotracer solution was performed on an Elysia-Raytest QC-Cubicle compact unit equipped with all devices for quality control. The in-house production of radiopharmaceuticals is regulated in the German Pharmaceuticals Act and the European Pharmacopeia. There are monographs for [^68^Ga]Ga-Octreotide (GALLIUM (68Ga) EDOTREOTIDE INJECTION, 2011) on which some of the product specifications were based:

- The radiochemical purity as determined by radio-TLC and radio-HPLC have to exceed > 97% and > 95%, respectively.
- The half-life of the product has to be 1.133 ± 0.1 h and was determined using an activimeter
- The nuclide purity has to exceed 99.999% and was determined by gamma spectroscopy at an energy of the γ-line to be 511 ± 70 keV
- The endotoxin level had to be below 35 EU/mL in a maximum application volume of 5 mL and was determined by an EndoSafe PTS
- The product has to be sterile.
- The pH value has to be between 3.0–4.0 and was determined by a reflection photometer.

The pH value was defined by the manufacturer of the kit. Additionally, a bubble-point test of the sterile filter was performed. Finally, the sterility was determined retrospective by taking an aliquot of [^68^Ga]Ga-NeoB within a class A environment and testing the probe on the next day externally.

### ^68^Ga-NeoB PET/CT imaging protocol

A lowdose-CT (Siemens Biograph mCT, Biograph 40 VA44A, 32 + 8-line-CT, PETsyngo software VG51C) and then early whole-body PET/CT images were acquired from vertex to mid thighs with 8 bed positions and 3-min emission scans per bed position at 60 min after intravenous administration of the [^68^Ga]Ga-NeoB of 1.5–2 MBq/kg (135–229 MBq) into the antecubital vein. Contrast-enhanced PET/CT studies were performed on a 40-slice PET/CT scanner with 80 ml arterial contrast (Imeron). A delayed, second abdominal PET image was acquired at 120 min p.i. of the [^68^Ga]Ga-NeoB. Two experienced nuclear medicine physicians manually drew regions of interest on the liver lesions for each image using 3-dimensional ellipsoid isocontouring with the assistance of the corresponding CT images. The results were expressed as SUV_mean_ and SUV_max._

## Results

### Validation of quality control methods under GMP conditions

Since the NeoB kit was in a clinical trial state, the production process had to be validated within the GMP environment by performing six independent [^68^Ga]Ga-NeoB syntheses which met the above mentioned criteria. The HPLC, TLC and edotoxin methods were validated for selectivity, precision, resolution, robustness, limit of determination and recovery, by adding aliquots of [^69^Ga]Ga-NeoB or free ^68^Ga to the [^68^Ga]Ga-NeoB batches. A summary of the validation can be found in Table 1. A complete validation report can be found in the supporting information. The validation report includes all significant results, the comparison with its specifications and the assessment of whether the specifications were met.

**Table 1:** Summary of the validation of the quality control methods fo [^68^Ga]Ga-NeoB

**Product identification – HPLC**			
***Selectivity***			
Specification	P*	result	9.85 min
Specification	S*: t(P) - t(S) = 0.05 ± 0.035 min	result	9.80 min
Specification	V*: t(P) - t(V) > 0.25 min	result	9.48 min
Specification	G*: t(P) - t(G) > 0.25 min	result	0.68 min
***Precision***			
Specification	**P**: mean + 3 s-value	result (*n* = 6)	9.73 ± 0.20 min
**Radiochemical puritiy – HPLC**			
***Resolution R between G and P***			
Specification	R > 1.5	result	R = 56.8
***Robustness***			
Specification	***P*** = 9.73 ± 0.20 min	result	9.77 min
***Limit of determination (S/N for 5 MBq/mL)***			
Specification	S/*N* > 10	result	37.9
***Recovery***			
Specification	W = 80–120%	result	87.3–92.7%
**Comparison between RCP HPLC and TLC**			
Variance	0.7%	result	HPLC 98.5% TLC 99.2%
**Product identification TLC**			
***Precision***			
Specification	**P** R_f_ = 0.6–0.9	result	R_f_ = 0.72
Specification	**G** R_f_ = 0–0.1	result	R_f_ = 0.04
Specification	**P**: mean + 3 s-value	result (n = 6)	R_f_ = 0.72 ± 0.08
***Robustness***			
Specification	***P*** = 0.72 ± 0.08 min	result	0.69 min
**Endotoxine test**			
Specification	‘pass’ < 2.5 EU/mL at 1:50	result (n = 6)	all ‘pass‘< 2.5 EU/mL

*P product [^68^Ga]Ga-NeoB; **S** non-radioactive standard [^69^Ga]Ga-NeoB; **V** precursor NeoB; **G** generator eluate pure ^68^Ga^3+^

All specifications were met so that the radiochemical purity of the product [^68^Ga]Ga-NeoB can be validly determined by HPLC and TLC. Under the HPLC method parameters and an injection volume of 10 μL, a product peak is obtained at a retention time of 9.73 ± 0.20 min. Under the TLC method parameters and a drop volume of 2 μL, a product peak with an R_*f*_ value of 0.72 ± 0.08 min is obtained. With endotoxin method parameters a valid endotoxin test with stable values is obtained.

### Validation runs for [^68^Ga]Ga-NeoB

**Fig. 1:**
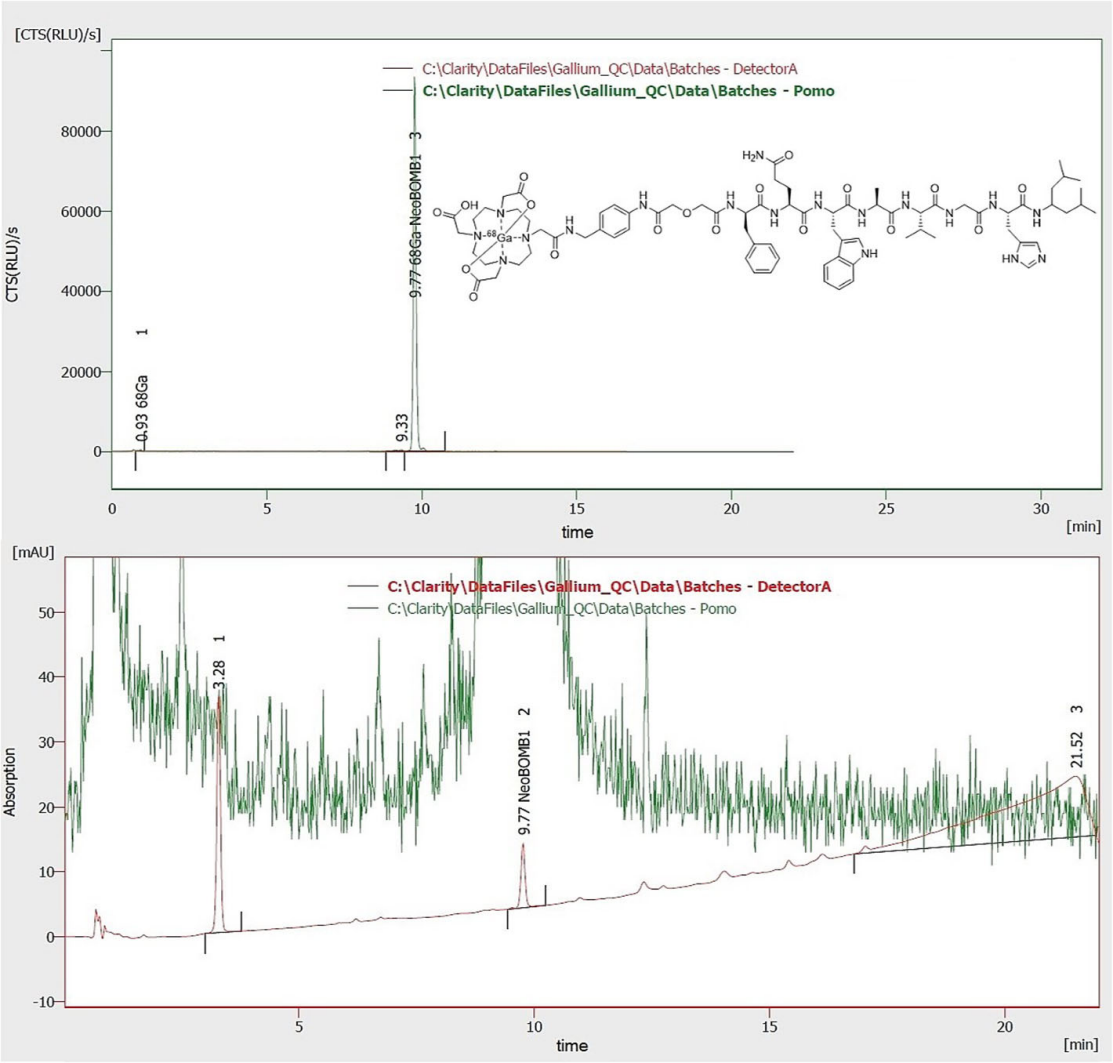
Representative (radio-)chromatogram of [^68^Ga]Ga-NeoB quality control. Green represents the activity signal and red the UV signal. The chemical structure of [^68^Ga]Ga-NeoB is in the upper chromatogram. The lower chromatogram shows a zoom of the upper chromatogram for determination of non-radioactive impurities

The results of the validation runs are summarized in Table 2. Runs with a different buffer amount were performed to evaluate and validate the possible effect of inaccuracies during the addition by different operators. The generator was eluted automatically using the mentioned GRP module equipped with an 20 mL syringe by pushing 0.1 M HCl (EZAG) through the generator (2 mL/min) and through a sterile filter for safety reason into the reaction vial containing the precursor for [^68^Ga]Ga-NeoB (Eder et al., 2014). The elution was performed with 5.5 mL 0.1 M HCl, resulting in 5.0 mL ^68^Ga-solution in the reaction vial and not less than 0.5 mL buffer solution (maximum 0.55 mL). In comparison to productions A (0.50 mL buffer solution) the insignificant higher amount (0.55 mL buffer solution) in productions B resulted in the same RCP using the TLC-method, but the RCP determined by HPLC demonstrated a slightly higher RCP (Fig. 1). The production-SOP for [^68^Ga]Ga-NeoB has to instruct to use a minimum amount of buffer of 0.50 mL. In conclusion, all six consecutive runs were inside of all the specifications yielding 712 ± 73 MBq of [^68^Ga]Ga-NeoB in a radiochemical yield of > 95%. The room conditions were proper and the devices are qualified for the production; thus the production process as carried out was demonstrated to be valid for patient production of [^68^Ga]Ga-NeoB. The product is at least stable for > 2 h. 700 MBq of [^68^Ga]Ga-NeoB decayed to ~ 175 MBq within 2 h. This corresponds to one patient dose. Longer times were therefore not testes.

**Table 2:** Results of the validation runs of [^68^Ga]Ga-NeoB

Run #	batch #			Reaction		Quality control			
	Buffer batch	buffer [ml]	HCl* [ml]	temp. time	yield** [MBq]	pH	radionuclide purity ^68^Ga-content	RCP: HPLC / TLC	Sterile
A-1	CT00516004 F03517002	0.50	5.0	95.1 °C 10 min	765	3.5	1.13 h > 99.999%	96.1% 99.0%	yes
A-2	CT00516004 F03517002	0.50	5.0	94.9 °C 9 min	716	3.5	1.12 h > 99.999%	96.4% 98.7%	yes
A-3	CT00516004 F03517002	0.50	5.0	95.1 °C 8 min	568***	3.4	1.12 h > 99.999%	97.0% 99.9%	yes
B-1	CT00516004 F03517002	0.55	5.0	95.0 °C 8 min	746	3.8	1.13 h > 99.999%	98.5% 99.2%	yes
B-2	CT00516004 F03517002	0.55	5.0	95.0 °C 10 min	756	3.8	1.12 h > 99.999%	97.0% 98.5%	yes
B-3	CT00516004 F03517002	0.55	5.0	95.2 °C 7 min	722	3.7	1.13 h > 99.999%	98.7% 99.4%	yes

*: Elution volume minus 0.5 mL dead volume of the tubing

**: measured 2–5 min after cool down

***: second generator elution of the day (first elution 2.5 h before)

Regarding toxicity of NeoBOMB1, two acute toxicity study in rats were performed. The first study assessed the toxicity of the peptide itself (crude NeoBOMB1), administered at 0.7 mg/kg or 1.4 mg/kg. No signs of toxicity were observed in this study. The second study assessed the toxicity of the peptide in formulation (including all the excipients that were included in the kit); in this case the tested dose was lower (0.25 mg/kg). No signs of toxicity were observed in this study like it is known for bombesin-1 and its derivatives (Gonzalez et al., 2008). Both studies were conducted in GLP-regulated environment.

### [^68^Ga]Ga-NeoB: clinical PET/CT investigations

For an implementation in clinical routine, three patients (2♀, 1♂, 51–77 a) with biopsy proven, metastatic GIST were examined with the GMP-produced [^68^Ga]Ga-NeoB via PET/CT for staging purposes after they had been treated by antiproliferative drug therapy (imatinib, sunitinib, regorafenib) followed by SIRT, IRE or MWA. Image acquisition, attenuation correction, fusion, reconstruction and post-processing were performed on a dedicated workstation. SUV_max_ was determined both on the initial whole-body and on the focused imaging later.

**Fig. 2:**
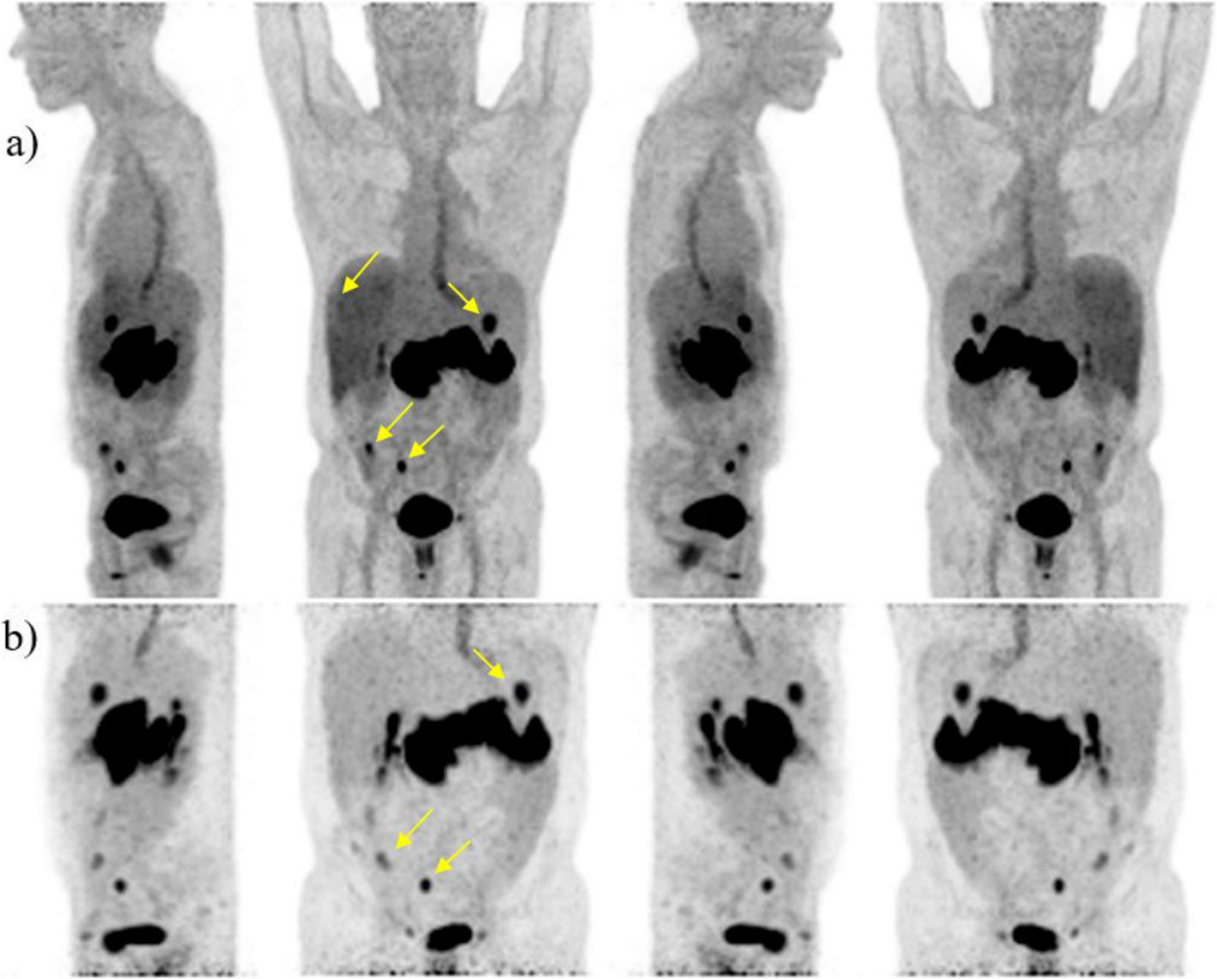
**a**) Maximum-intensity-projections (MIP) of (♂, 57) 1 h p.i. **b**) 2 h p.i. left, front, right, behind

Patient #1 (male, 57 years old, small bowel GIST with peritoneal and progressive liver metastases after 3rd line therapy carrying an exon 11 and a 2ndary exon 17 mutation) received [^18^F]FDG PET/CT for staging and two of the progressive liver metastases in segment VII and segment II/III were depicted. The patient underwent subsequent SIRT therapy. Four months after therapy the patient received [^68^Ga]Ga-NeoB (229 MBq) for follow-up staging (Fig. 2) with low accumulation in the lesion in liver segment VII (SUV_max_ early of 1.4, SUV_max_ late of 3.3), but with persistence of the radiotracer in liver segment II/III (SUV_max_ early of 6.3, SUV_max_ late of 16.1), indicating still vital tumor tissue. Thus, the patient again underwent IRE on the left lobe (segment II/III). In comparison to the preliminary examination with [^18^F]FDG, two newly occurring demarcated peritoneal metastases in the right hemiabdomen with increased nuclide uptake (SUV_max_ early of 3.3 and 5.7, SUV_max_ late 6.4 and 17.9, respectively) were detected with [^68^Ga]Ga-NeoB. In addition, physiological distribution of [^68^Ga]Ga-NeoB was found in the study area. At a three-month [^18^F]FDG PET/CT follow-up the patient showed further progressive disease.

**Fig. 3:**
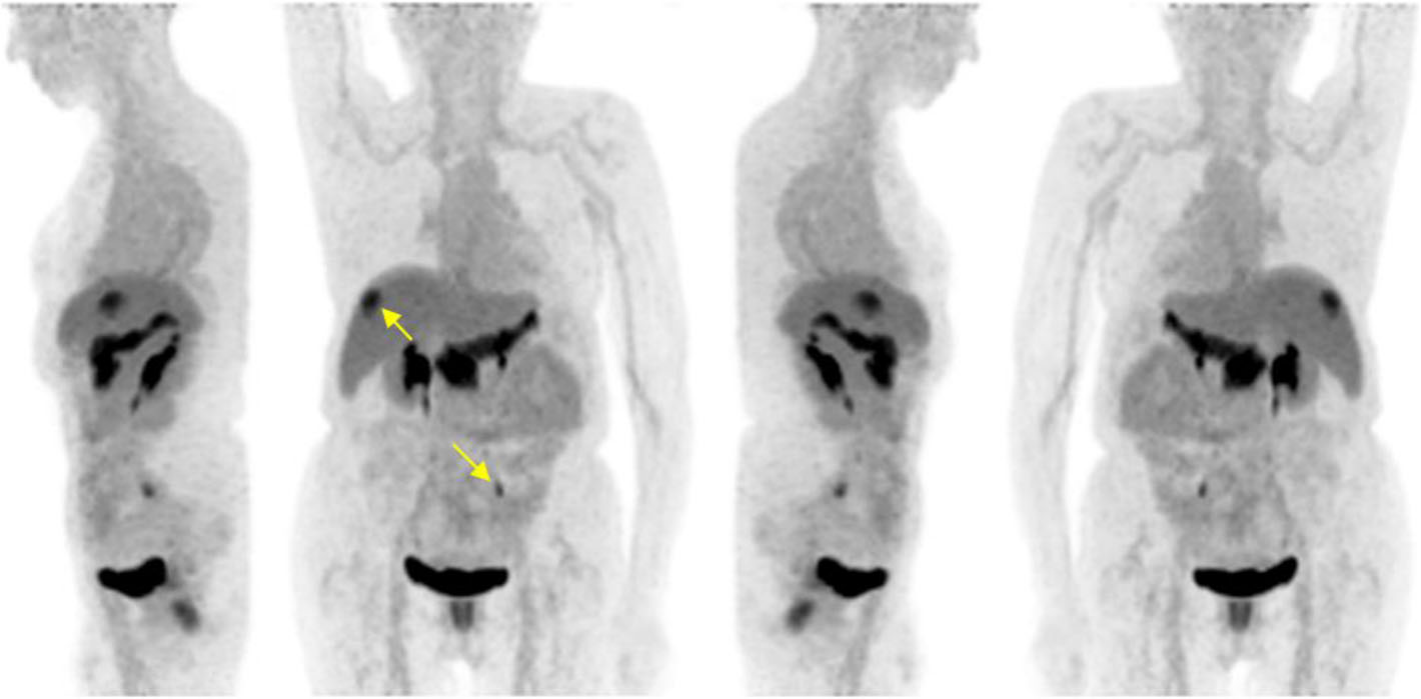
MIP of (♀, 77) 1 h p.i. b) 2 h p.i. left, front, right, behind

Patient #2 (female, 77 years old, small bowel GIST with progressive peritoneal and soft tissue metastases under third line therapy carrying an exon 11 and two different secondary mutations in exon 13) received [^68^Ga]Ga-NeoB (202 MBq) PET/CT for staging. A previously unknown isolated hypodense liver lesion in segment VII (SUV_max_ early of 11.2, SUV_max_ late of 16.6) was found (Fig. 3). Additionally, an abdominal wall metastasis in the left lower abdomen was found, which was not previously known from a three-month preliminary [^18^F]FDG PET/CT and was later proven histologically by surgical resection. In addition, physiological distribution of [^68^Ga]Ga-NeoB was visible in the study area. The single metastasis in liver segment VII was treated by IRE and no lesions were found by magnetic resonance imaging (MRI) follow-up 11 months later. Therefore, the patient was considered to have experienced a complete response.

**Fig. 4:**
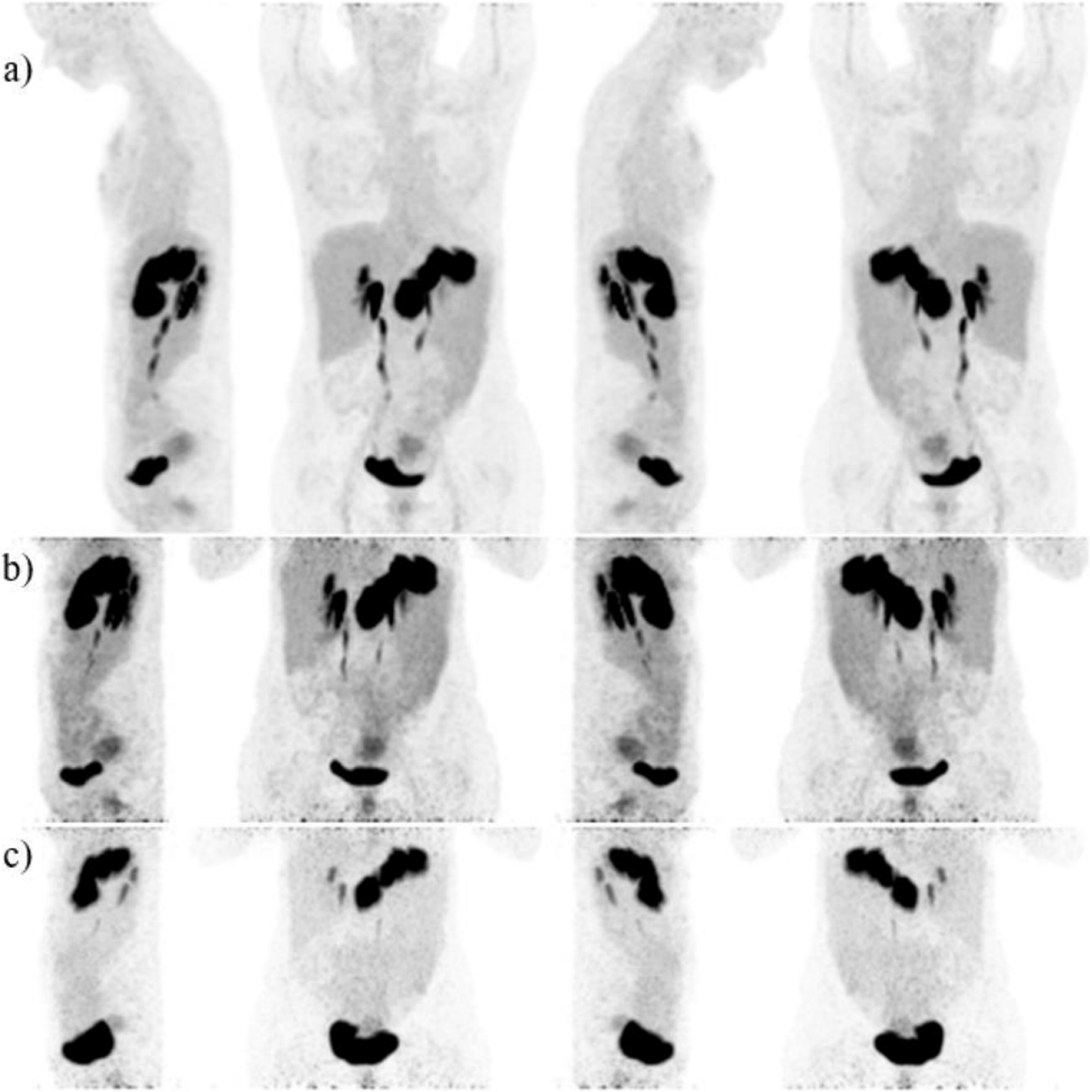
**a**) MIP of (♂, 57) 1 h p.i. **b**) 2 h p.i., and **c**) 3 h p.i. left, front, right, behind

Patient #3 (female, 51 years old, multiple progressive hepatic metastases of GIST of the stomach carrying a D842V mutation in PDGFRa and being pretreated as treated by SIRT and microwave ablation at previously known hepatic metastases. Three months later the patient received [^68^Ga]Ga-NeoB (135 MBq) PET/CT for staging (Fig. 4). An inhomogeneous, flat tracer accumulation within the uterine cavity with decrease of uptake in the temporal course (SUV_max_ 60 min p.i. of 26.5, SUV_max_ 120 min p.i. of 7.1, SUV_max_ 180 min p.i. of 6) was found, most likely a physiological enrichment. In addition, physiological radiopharmaceutical distribution in the study area was observed. However, there was no pathologically increased uptake of [^68^Ga]Ga-NeoB in the known hepatic metastatic lesions which had an unchanged morphology in comparison to the three-month previously performed [^18^F]FDG PET/CT. No tumor uptake and no new metastases were found in the region of interest also at later time-points indicating a stable disease. In the 120 min p.i. images, an increased nuclide uptake was found in the region of the gall bladder neck (Fig. 4b). However, after fatty eating, there was no correlation in 180 min p.i. images (Fig. 4c). An additional MRI follow-up examination six months after therapy was performed, which confirmed a stable disease.

## Discussion

Six validation runs for [^68^Ga]Ga-NeoB were performed and proved a stable method and quality control for the GMP-compliant production in clinic following the most recent guidelines (Todde et al., 2017; Gillings et al., 2020). The specificity of the HPLC and TLC methods were proven. For a detailed method validation see the supporting information. No issues in synthesis or quality of [^68^Ga]Ga-NeoB were found. Therefore, the radiotracer was ready to be used safely for PET diagnosis.

[^68^Ga]Ga-NeoB was introduced in the clinical routine in three patients with different diagnostic scenarios. In the [^68^Ga]Ga-NeoB studies two new lesions in patient 1 and one more lesion in patient 2 were detected, which were not previously known. These results suggest that [^68^Ga]Ga-NeoB imaging may be an additional useful tool for detection of new lesions or metastases compared to conventional [^18^F]FDG PET/CT or MRI with unclear outcome. The uptake of the radiotracer in the newly detected metastases is mainly related to the overexpression of GRPR, which was histologically documented in our cases. However, patient #3 was stable between therapy and [^68^Ga]Ga-NeoB PET/CT follow-up and the patient had a complete response.

Additionally, patient #1 was assessed with a progressive disease because of increased high uptake in segment II/III and newly detected lesions, whereas the lesion in segment VII was confirmed stable showing no [^68^Ga]Ga-NeoB uptake after SIRT therapy. The reason for the higher uptake in segment II/III could be a possible increased GRPR expression in growing GIST lesions or in metabolic active phases (Gruber et al., 2020). Although patient #1 underwent SIRT and IRE, he showed a progressive disease in the follow-up examination after four months. It could be suggested, that endoradiotherapy with [^177^Lu]Lu/[^225^Ac]Ac-NeoB would be an additional, more specific therapeutic option to SIRT, IRE or MWA in patients with high GRPR-expressing lesions in multiple organs. However, it should be taken into account that if tumorous lesions known from morphologic imaging do not show a [^68^Ga]Ga-NeoB uptake, this might also be related to low GRPR expression (Gruber et al., 2020).

It is well known that GIST have a wide spectrum of mutations, some of it with very low incidence (Lasota et al., 2006). Although 70–75% of GIST harbor imatinib-sensitive mutations of KIT (Linch et al., 2013), secondary resistances are often acquired within 2 years (Breeman et al., 1999; Gruber et al., 2020; Takahashi et al., 2017; Wardelmann et al., 2005). The mechanisms of primary and secondary therapy resistance in GIST are not completely understood. It could be speculated that patients showing no uptake in [^68^Ga]Ga-NeoB PET/CT in lesions known from conventional [^18^F]FDG PET/CT or MRI imaging might have progressive disease and therapy resistance due to mutations affecting GRPRs. [^68^Ga]Ga-NeoB imaging could be a useful additional modality regarding monitoring of the effectiveness of a therapy of GIST patients and assisting to choose the type of therapy.

## Conclusion

[^68^Ga]Ga-NeoB was successfully integrated in the clinical diagnostic procedures by routine production under GMP conditions. The application of the tracer could be introduced to the care of patients with GIST metastases in the liver and abdominal cavity. With the combination of PET/CT it was possible to evaluate therapy response in GIST patients with liver metastases. Patients who underwent [^18^F]FDG PET/CT with an inconclusive result on therapeutic response could benefit from an additional diagnostic approach with [^68^Ga]Ga-NeoB for characterization of GRPR-expressing tumors. In the future, ^177^Lu/^225^Ac-labeled NeoB may also be used for endoradiotherapy of high GRPR-expressing tumors. However, further clinical diagnostic studies are warranted prior to a therapeutic approach.

## Supplementary Information

**Additional file 1.**

## Data Availability

The data used and analysed during the current study are available from the corresponding author on reasonable request.
