# Supplementary material for: GMP-compliant production of [68Ga]Ga-NeoB for positron emission tomography imaging of patients with gastrointestinal stromal tumor
Source: EJNMMI Radiopharm Chem. 2021 Jul 6;6:22. doi: 10.1186/s41181-021-00137-w (PMC8260665; doi:10.1186/s41181-021-00137-w)
Supplement: Supplementary file 1 — Additional file 1. [file 41181_2021_137_MOESM1_ESM.doc]

**Supporting Information**

**GMP-compliant production of [^68^Ga]Ga-NeoB for positron emission tomography imaging of patients with gastrointestinal stromal tumor**

**Marc Pretze^1,2^*, Laura Reffert^1^,** **Steffen Diehl^3^, Stefan O. Schönberg^3^, Carmen Wängler,^4^ Peter Hohenberger^5^, Björn Wängler^2^***

**Validation of the quality control methods for [^68^Ga]Ga-NeoB**

For the validation of quality control of [^68^Ga]Ga-NeoB six independent batches were produced and used for method validation of the GMP-compliant quality control following the most recent guidelines (Todde et al. EJNMMI Radiopharm Chem 2017 and Gillings et al. EJNNMI Radiopharm Chem 2020).

**Definitions and shortcuts**

AAA Advanced Accelerator Applications

NeoB NeoBOMB1

EL Eluent

MeCN Acetonitrile

TFA Trifluoroacetat

RCP Radiochemical purity

Rt Retention time

w Peak width

s Standard deviation

P [^68^Ga]Ga-NeoB

S [^69^Ga]Ga-NeoB

V NeoBOMB1

G ^68^Ga^3+^

MV Mean value

*Product identification*

1. Selectivity:

Selectivity is tested by UV detection of the non-radioactive compound **V** and the radioactive **G** compared to the product **P** using a radio chromatogram. Selectivity is given if the difference in retention times is greater than the ±3s value of P. The selectivity is additionally determined by comparison with TLC analysis.

Here, the retention time between **P** and **S** is also checked (taking into account the dead time between UV and radio detector). For an unambiguous assignment of **S** to **P**, the difference of the retention time is equal to the dead time between UV and radioactivity detector 0.07±0.035min (line ID= 0.1mm, 25cm line, flow 1 mL/min => 0.07min).

1. Precision:

The precision describes the random scatter of the analysis results. For this purpose, the analysis of **P** is performed 6 times. The measured retention times of **P** are used to calculate the standard deviation. The specification of the scatter of the retention time results from the ±3s value, which according to Gauss corresponds to a probability of 99.80% that the sought value is to be found in this range.

*Radiochemical purity*

1. Resolution between **G** and **P**

For quantitative determination of the radiochemical purity, a resolution of > 1.5 must be given. The resolution R is calculated as follows:

1.18 x (Rt_2_ - Rt_1_) Rt Retention time

R = ————————

w0.5_2_ + w0.5_1_ w0.5 Peak width at half height

10 µL of **P** is analyzed by the method. If no impurity **G** is present, the sample **P** is mixed with **G** in a ratio of 1:1 (v/v) and 20 µL is analyzed by HLPC. The resulting retention times and peak widths are taken from the integrated chromatogram and used to calculate the resolution.

1. Robustness:

The robustness of the method is given if the final result does not change despite changed framework conditions. The main influencing parameters are the ambient temperature and the pH value of the solution. In the case of the analysis of [^68^Ga)Ga-NeoB, the analysis takes place under controlled ambient temperature (18–25°C). In this temperature range, as known from literature, the influence on the separation is negligible. Due to the buffer addition at the end of the preparation, a constant pH range of the ready-to-inject solution is obtained, which is checked and documented during QC. Therefore, in this test the robustness (recovery of the retention time) is checked by an artificially induced change of the pH value by addition of e.g. an acidic solution (10 µL **P** pH 3–5 + 10 µL **G** pH 1.8 - injection of 20 µL).

1. Limit of determination:

The RCP is calculated using the area of the integrated radioactive product peak relative to the sum of all radioactive areas of the chromatogram. For this calculation to be meaningful, the product peak must have a height greater than 10 times the background (corresponding to the limit of quantitation). The peak area of the radio-chromatogram is halved according to the half-life of the corresponding nuclide (T_1/2_ Ga-68 = 68 min). The analysis of the ready-to-inject solution is performed immediately after completion of the preparation. After completion of all quality controls, at least 100 MBq in 5 mL must be available for application to the patient. An aliquot of 10 µL of this solution contains the required amount of activity to generate a unique peak. For this detection, the injection solution is diluted to 5 MBq/mL (less than 0.05 MBq/10 µL; equivalent to 25 MBq in 5 mL) with water. 10 µL of this solution is analyzed by radio-HPLC and the signal-to-noise ratio is determined.

2H H…Height of peaks (Peak maximum - extrapolated baseline)

S/N = h h… Area with width of at least 5 times the peak width

1. Recovery rate:

In the case of radiochemical analysis, the recovery rate considers the aspect that radioactive components could possibly be retained by the column material. Therefore, it is tested how much activity of the injected activity can be recovered in the waste after the end of the analysis. This test is performed in a triple determination:

- only with **P**

- with **G** only

- combination of **G** and **P**

The activity of the drawn up injection syringe is determined on the activimeter before and after injection in order to calculate the actual injected activity. The EL amount of the entire run is collected and measured. The obtained waste fraction is decay corrected to the time of injection and the two values "injected activity" (At_0_) and "recovered activity" (At_w_) are put into relation:

At_w_

W (%) = At_0_ x 100

Specification: The recovery rate W should be 80–120% (due to the high measurement error range of the activimeter for the reference measurement, the specification has been set in a wider range).

1. Linearity:

The range in which meaningful calculations of radiochemical purity may take place results from the measuring range of the detector system. This has already been determined in the course of the functional qualification for gallium-68. Overloading the detector would lead to a disproportionate dead time correction and thus falsify the result. The maximum injectable amount of activity results from the theoretically maximum obtainable activity of the product, which in turn is limited by the maximum obtained starting activity by the ^68^Ge/^68^Ga-generator and the time between the start and the end of the synthesis (decay of the radioactivity). The resulting maximum amount of activity in 10 µL of product solution injected for analysis is, in purely arithmetical terms, below the amount of activity that would overload the radioactivity detector and therefore does not need to be checked again.

1. Comparison with radio-TLC method:

The radiochemical purity can be determined alternatively to the radio-HPLC method by means of the radio-TLC method. If both chromatographic methods show the same results independent of the running medium and stationary phase, it can be assumed that the radio HPLC method described is suitable for determining the radiochemical purity. The data for this can be found in the validation report for the radio TLC method (see chap. 2).

*Chemical purity*

According to the manufacturer (AAA), there are 45–55 µg of NeoB precursor in the KIT. Thus, after manufacturing, the final product has a maximum final concentration of 11 µg/mL of precursor and cold product.

No own toxicity data are available from the KIT manufacturer, but he refers to the publication Gonzalez et al. 2008, according to which bombesin-1 and its derivatives have no toxicity either in the applied concentration range or at significantly higher concentrations. Therefore, no quantification of the precursor or cold product is performed in the quality control process. In the course of quality control, the UV chromatogram is qualitatively assessed with respect to impurities.

**1. Validation protocol HPLC**

The validation protocol contains results, chromatograms, calculations and, if necessary, explanatory supplements for the individual test methods. The collected results are summarized in the following validation report.

*Reagents*

NeoB precursor: AAA , Lot: not specified, c = 0.5 mg/mL

[^69^Ga]NeoBOMB1: AAA, Lot: 170810, 1000 ppm

Water: TracePur, Merck, Lot: Z0349973523

*Method parameter*

1. HPLC system: Shimadzu with quaternary pump system, degasser, mixing chamber, UV-VIS detector, manual injector, radio detector, system controller and Clarity software (the exact data and properties of the individual components can be taken from the qualification documents of Elysia)
2. HPLC column: LiChrospher 100 RP18 5µ EC: 125 x 3 mm
3. Eluent: **A**: MeCN + 0.1% TFA

**B**: H_2_0 + 0.1% TFA

(all used EL have HPLC-grade purity)

1. Flow rate: 1 mL/min.
2. Gradient: 10%–90% **A** in 20 min, followed by 5 min 90% **A**
3. Detection: UV at 254 nm, NaI crystal radio detector (for PET-nuclides)

*Probe preparation*

- [^68^Ga]Ga-NeoB (**P**): The analysis of the product **P**, requires the synthesis of the precursor molecule NeoB **V** with gallium-68. The product obtained corresponds to an injection-ready solution and is analyzed directly (without further workup) by HPLC. Characterization of this solution is additionally performed by other methods such as radio-TLC, pH analysis, half-life control and gamma spectroscopy.
- [^69^Ga]Ga-NeoB (**S**): The cold standard is injected with either pure or mixed with the **P** in a 1:1 ratio. (storage in refrigerator)
- NeoBOMB1 (**V**): The precursor is injected with either neat or mixed with the P in a 1:1 ratio. (Storage in refrigerator)
- ^68^Ga^3+^ (**G**): The ^68^Ga-eluate contains 0.1 M HCl in water. ^68^Ga^3+^ is eluted with the runner front (short Rt) due to its polarity under the selected conditions.

**P** and **G** can only be analyzed by radio detector, while **S** and **V** can only be detected in the UV chromatogram.

In a preliminary experiment, the HPLC conditions for ^68^Ga-PSMA/TATE were initially selected. However, [^68^Ga]Ga-NeoB is much less polar and has a much too long retention time with the gradient for ^68^Ga-PSMA/TATE (10–50% **A** in 20min, then 5 min **A** at 50%) to be analyzed in time (see first chromatogram, Figure 1). Therefore, the HPLC conditions were adjusted (gradient: 10–90% **A** in 20 min, then 5 min **A** at 90%) (see second chromatogram, Figure 1).

Figure 1: (Radio)-chromatogram of [^68^Ga]Ga-NeoB with different eluent gradients.

# Protocol: Selectivity

Selectivity testing was performed by co-injection of product solution **P** with either standard cold compound **S**, precursor **V**, or pure ^68^Ga^3+^ (**G**).

Table 1:

| **Peak** | **S R_t_ UV [min]** | **P R_t_ Radio [min]** | **Difference R_t(Radio - UV)_ [min]** |
| --- | --- | --- | --- |
| S <->P | 9.80 | 9.85 | 0.05 |
| **Peak** | **V R_t_ UV [min]** | **P R_t_ Radio [min]** | **Difference R_t(Radio - UV)_ [min]** |
| V <->P | 9.48 | 9.77 | 0.29 |
| **Peak** | **G R_t_ Radio [min]** | **P R_t_ Radio [min]** | **Difference R_t(Radio - UV)_ [min]** |
| G <->P | 0.68 | 9.82 | 9.14 |

The difference between the retention times of **P** and **G** is greater than the 3s value of 0.20 min (see 5.5). The difference between **P** and **V** is also greater than the 3s value of 0.20 min. The difference between **S** and **P** was within the specification of 0.07 ± 0.035 min. Thus, all compounds could be clearly identified from each other or **P** and **S** could be assigned to each other (UV + radio, Figure 2) under compliance with all specifications. In addition, the area of 20 µg [^69^Ga]Ga-NeoB was determined at 456 mAu × sec. The values of the product should generally be below this value, which could be confirmed in the subsequent measurements.

Figure 2. HPLC chromatogram - detail section: showing the similar retention times of **P** and **S** upon co-injection of 20 µg [^69^Ga]Ga-NeoB.

# Protocol: Precision

Based on the study of 6 independent batches, the dispersion of the retention time of the product peaks 3s = 0.20 min was calculated around the mean value 9.73 min with a confidence level of 99.80% (table 2).

Table 2: Retention times of [^68^Ga]Ga-NeoB for six independent batches.

| **#** | **R_t_**  **[min]** |
| --- | --- |
| 1 | 9.70 |
| 2 | 9.70 |
| 3 | 9.70 |
| 4 | 9.68 |
| 5 | 9.77 |
| 6 | 9.85 |
| **MV 1 [min]** | 9.73 |
| **s [min]** | 0.07 |
| **3s [min]** | 0.20 |

**Protocol: Resolution**

To calculate the resolution between free ^68^Ga^3+^ **G** and the product **P**, the peaks closest to each other (peak no. 1 and 2, table 3, figure 3) are used.

- Calculation step 1: 1.18 × (9.43 - 0.68) = 10.79
- Calculation step 2: 10.79 ÷ (0.12 + 0.07) = **56.79**

The specificity of the separation has been determined by the resolution R = 56.79, which is greater than the minimum required resolution of 1.5.

Table 3: Peak identification for calculating the resolution

| **Nr.** | **Identification** | **R_t_ [min]** | **Peak width w0.5 at half height [min]** |
| --- | --- | --- | --- |
| 1 | ^68^Ga^3+^ (**G**) Peak 1 | 0.68 | 0.07 |
| 2 | [^68^Ga]Ga-NeoB (**P**) Peak 2 | 9.82 | 0.12 |

Figure 3: HPLC chromatogram of a co-injection of **G** and **P**.

# Protocol: Robustness

The retention times of the product peaks meet the specification Rt = 9.73 ± 0.07 min regardless of sample preparation. The deviations of the measured retention times of the ready-to-inject solution and the solution diluted 1:1 with 0.1M HCl (generator eluate solution) from the mean value are within the maximum deviation of 3s value. Thus, the robustness of the method is given.

Table 4: Values from chromatogram in figure 4.

| **Peak** | **P from injection solution**  **Rt [min]** | **Deviation from MV [min]** | **Dilution 1:1 with 0.1 M HCl**  **Rt [min]** | **Deviation from MV [min]** |
| --- | --- | --- | --- | --- |
| 1 | 9.73 | 0.04 | 9.77 | 0,04 |

Figure 4: HPLC chromatogram of a 1:1 mixture of 0.1 M HCl generator eluate **G** and **P**.

# Protocol: Limit of determination

The background noise was determined in a period of 12 to 20 min. (corresponding to a period of 8 min.) and was 19.0654 [CTS(RLU)/s]. This period corresponds to more than 5 times the peak width of the product peak. The injection activity of 0.022 MBq in 10 µL corresponds to 2.2 MBq/mL. The required specification that the peak height is 10 times higher than the noise (= definition of the limit of quantification) was met for the product peak (table 5 and figure 5).

Table 5: Values from chromatrogram in figure 5.

| **Peak** | **R_t_ [min]** | **Peak height [CTS(RLU)/s]** | **Peak height to noise ratio (S/N)** |
| --- | --- | --- | --- |
| 1 | 9.88 | 723 | 37.92 |

Figure 5: HPLC chromatogram showing the S/N ratio when injecting small amounts of activity (0.022 MBq in 10 µL).

# Protocol: Recovery rate

The measurements of injected activity and activity in the waste vessel were performed using an Isomed 2010 dose calibrator (activimeter).

The measurement errors of the instrument are:

> 0.400 MBq ±5%

< 0.400 MBq ±0.020 MBq (given in % relative to the measured activity).

All recoveries obtained are within the measurement range of the activimeter and meet the specification of a recovery in the range of 80–120% (table 6).

Table 6:

| **substance** | **injected activity**  **[MBq]** | **measured activity**  **[MBq]** | **recovery rate (decay-corrected) [%]** | **measuring error range activimeter [%]** |
| --- | --- | --- | --- | --- |
| **P** | 0.022 | 0.015 | 92.7 | 40 – 160 |
| **P + S** | 0.345 | 0.227 | 89.5 | 81.6 – 97.3 |
| **P + V** | 0.752 | 0.524 | 87.3 | 83.0 – 91.6 |

Protocol: Comparison with the radio-TLC

For the validation of the TLC method, see 2. Validation TLC

The specification that the product solution must have a radiochemical purity of >95% by both methods was met in both cases (table 7). In addition, a product sample spiked with ^68^Ga^3+^ was analyzed by both radio-TLC (see table 8 and figure 6) and radio-HPLC (see Figure 3) and their relative integrals (areas) were compared. A difference in the ^68^Ga^3+^ content of 5.7% was found. The difference is mainly due to the fact that the HPLC column absorbs ^68^Ga-colloid completely and small amounts of the free ^68^Ga^3+^ (this is not significant for amounts <5%) and is therefore not detected.

Table 7: Comparison of the same ready-to-inject solution showed the following radiochemical purity.

| **Peak (substance)** | **portion HPLC [%]** | **portion TLC [%]** | **Deviation [%]** |
| --- | --- | --- | --- |
| ^68^Ga^3+^ | 0.93 | 0.77 | 0.73 |
| [^68^Ga]Ga-NeoB | 98.5 | 99.23 |
| other | 1.1 | 0 |

Table 8: TLC-values of a mixture of G and P.

| **Peak/ substance** | **portion HPLC [%]** | **portion TLC [%]** | **Abweichung [%]** |
| --- | --- | --- | --- |
| ^68^Ga^3+^ | 50.4 | 56.1 | 5.7 |
| [^68^Ga]Ga-NeoB | 49.6 | 43.9 |

Figure 6: Radio-TLC chromatogram of a mixture of **G** and **P**: region 1 (Rf = 0.09) shows ^68^Ga^3+^ **G**, region 2 (Rf = 0.74) shows product **P**. The verification of the two regions is described in the corresponding method validation (TLC).

**Validation of the evaluation method**

1. **Plan**

The automatic evaluation method significantly saves time when performing quality control and makes it easier for employees to perform the evaluation. However, since a 'threshold' must be set for the automatic evaluation, the automatic evaluation must also be validated.

The validation of the evaluation method can be done computationally if the resulting error in radiochemical purity by the automatic evaluation is less than 0.25%.

1. **Result**

In the automatic evaluation, the threshold was adjusted to integrate all peaks that have a peak height 10 times that of the background noise (= 19.07 CTS/s × 10). If the peak of free ^68^Ga^3+^ has just 10 times the background noise, this leads to a radiochemical purity of [^68^Ga]Ga-NeoB of 99.8% (191 CTS/s ÷ 94958 CTS/s = 0.002 = 0.2%) (100% - 0.2% = 99.8%) for a normal production. This means that an error of 0.2% in radiochemical purity must be expected, since free ^68^Ga^3+^ below 0.2% is not automatically cointegrated. However, this error is negligible.

Mathematically, 2442 CTS/s are obtained from a calibration curve for a minimum injection quantity of 0.05 MBq/10 µL (= 5 MBq/mL) (figure 7). This corresponds to 128 times the peak height of the background noise or about 13 times the peak height of the 'threshold' for automatic integration. Thus, even the minimum injection amount is still clearly detected and evaluated by the automatic integration.


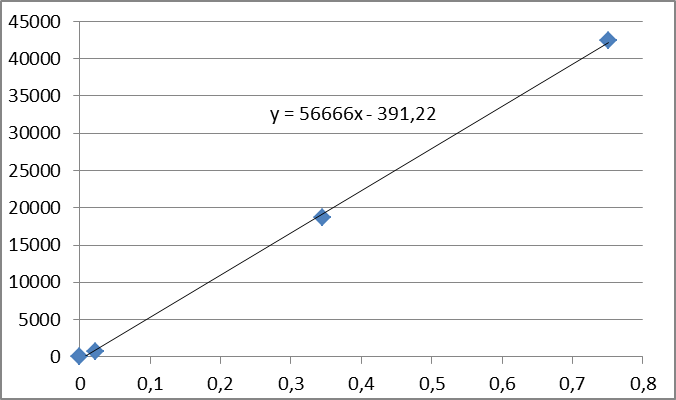


Values for calibration curve

| MBq | CTS/s |
| --- | --- |
| 0.752 | 42464 |
| 0.345 | 18638 |
| 0.022 | 723 |
| 0 | 19 |

Figure 7: Calibration curve for peak area to activity

If the RCP is near the limit of 95%, i.e. corresponds exactly to the specification, the peak integration in the radiochromatogram (POMO, blue/green) must be adjusted manually (delete/add/name peaks) and additionally the UV channel (red) must be checked for impurities.

The error of the automatic evaluation is a maximum of 0.2% and is thus below the specification of 0.25%. The automatic evaluation thus provides valid results.

**2. Validation TLC**

# Plan

The reason for changing the running medium as suggested by AAA is the practical application and integration into the running GMP operation in the NUK. Since two ^68^Ga-products were already being investigated with the same running medium (1 M ammonium acetate:methanol V:V = 1:1), the aim was to validate whether this running medium also ensures separation for the differentiation of ^68^Ga^3+^ and [^68^Ga]Ga-NeoB.

# Protocol

The running medium proposed by AAA (5 M ammonium acetate:methanol:water V:V=1:7:2) should result in a specification of Rf values as follows:

- colloidal ^68^Ga^3+^ = 0–0.1

- [^68^Ga]Ga-NeoB = 0.6–0.9

Here, 5 µL of the final [^68^Ga]Ga-NeoB solution should be applied to an ITLC strip.

The Rf-value of free ^68^Ga^3+^ has a mean value of 0.06 and a scatter of 0.03 (table 9). The Rf-value of [^68^Ga]Ga-NeoB has a mean value of 0.72 and a scatter of 0.08. The 3s-values show that a clear separation is always guaranteed. The method is equivalent to the method proposed by AAA and thus valid for distinguishing ^68^Ga^3+^ from [^68^Ga]Ga-NeoB within the quality control of [^68^Ga]Ga-NeoB.

Table 8: Rf values with the new running medium 1 M ammonium acetate:methanol V:V=1:1 (6 measurements)

| **#** | **R_f_ ^68^Ga^3+^** | **R_f_ [^68^Ga]Ga-NeoB** |
| --- | --- | --- |
| 1 | 0.04 | 0.72 |
| 2 | 0.09 | 0.74 |
| 3 | 0.08 | 0.87 |
| 4 | 0.05 | 0.63 |
| 5 | 0.04 | 0.69 |
| 6 | 0.01 | 0.69 |
| **MV 1** | 0.06 | 0.72 |
| **s** | 0.03 | 0.08 |
| **3×s** | 0.08 | 0.24 |

**Protocol: Robustness**

The Rf values of the product peaks meet the specification Rf = 0.72 ± 0.08 min regardless of sample preparation. The deviations of the measured retention times of the ready-to-inject solution and the solution diluted 1:1 with 0.1 M HCl (generator eluate solution) from the mean value are within the maximum deviation of 3s (table 10, figure 8 and 9). Thus, the robustness of the method is given.

Table 9: Values from chromatrogram in figure 8.

| **Peak** | **P from injection solution**  **Rf** | **Deviation from MV** | **Dilution 1:1 with 0.1 M HCl**  **R_f_** | **Deviation from MV** |
| --- | --- | --- | --- | --- |
| 1 | 0.72 | 0.00 | 0.69 | 0.03 |

Figure 8: Thin layer chromatogram of the injection solution of [^68^Ga]Ga-NeoB **P** (Rf = 0.72) and **G** (Rf = 0.04).

Figure 9: Thin layer chromatogram of a 1:1 mixture of 0.1 M HCl generator eluate **G** and

**Validation of endotoxintest for [^68^Ga]Ga-NeoB**

When using the standard dilution of 1:20 for the endotoxin tests with the product solution of [^68^Ga]Ga-NeoB, a valid test result could not be obtained permanently (figure 10). The acid buffered solution with a higher salt content did not reliably detect the positive control.

At a dilution of 1:50 (980 µL water + 20 µL [^68^Ga]Ga-NeoB), this effect could be avoided and the device-internal verification of the suitability of the test was positive. All other 4 endotoxin tests of the validation syntheses also showed the suitability of the test for the [^68^Ga]Ga-NeoB product solution at a dilution of 1:50.

Thus, the dilution 1:50 in the [^68^Ga]Ga-NeoB quality control can be considered valid for the performance of the endotoxin test.

Figure 10: Endotoxin tests on the same batch of NEOB at dilutions of 1:20 (fail) and 1:50 (pass).

**3. Validation report**

The validation report includes all significant results shown in table 11, the comparison with its specifications and the assessment of whether the specifications were met.

Table 10: Summary of the validation of the quality control methods fo [^68^Ga]Ga-NeoB.

| **Product identification - HPLC** | | | |
| --- | --- | --- | --- |
| ***Selectivity*** | | | |
| Specification | **P*** | result | 9.85 min |
| Specification | **S***: t(P) - t(S) = 0.05 ± 0.035 min | result | 9.80 min |
| Specification | **V***: t(P) - t(V) > 0.25min | result | 9.48 min |
| Specification | **G***: t(P) - t(G) > 0.25min | result | 0.68 min |
| ***Precision*** | | | |
| Specification | **P** : mean + 3s-value | result (n = 6) | 9.73 ± 0.20 min |
| **Radiochemical puritiy – HPLC** | | | |
| ***Resolution R between G and P*** | | | |
| Specification | R > 1.5 | result | R = 56.8 |
| ***Robustness*** | | | |
| Specification | **P** = 9.73 ± 0.20 min | result | 9.77 min |
| ***Limit of determination (S/N for 5 MBq/mL)*** | | | |
| Specification | S/N > 10 | result | 37.9 |
| ***Recovery*** | | | |
| Specification | W = 80–120% | result | 87.3–92.7% |
| **Comparison between RCP HPLC and TLC** | | | |
| Variance | 0.7% | result | HPLC 98.5% TLC 99.2% |
| **Product identification TLC** | | | |
| ***Precision*** | | | |
| Specification | **P** R_f_ = 0.6–0.9 | result | R_f_ = 0.72 |
| Specification | **G** R_f_ = 0–0.1 | result | R_f_ = 0.04 |
| Specification | **P** : mean + 3s-value | result (n = 6) | R_f_ = 0.72 ± 0.08 |
| ***Robustness*** | | | |
| Specification | **P** = 0.72 ± 0.08 min | result | 0.69 min |
| **Endotoxine test** | | | |
| Specification | ‘pass’ <2.5 EU/mL at 1:50 | result (n = 6) | all ‘pass‘ <2.5 EU/mL |

***P** product [^68^Ga]Ga-NeoB; **S** non-radioactive standard [^69^Ga]Ga-NeoB; **V** precursor NeoB; **G** generator eluate pure ^68^Ga^3+^

All specifications were met so that the radiochemical purity of the product [^68^Ga]Ga-NeoB can be validly determined by HPLC and TLC. Under the HPLC method parameters and an injection volume of 10 µL, a product peak is obtained at a retention time of 9.73 ± 0.20 min. Under the TLC method parameters and a drop volume of 2 µL, a product peak with an R*_f_* value of 0.72 ± 0.08 min is obtained. With endotoxin method parameters a valid endotoxin test with stable values is obtained.
